# Secondary Structures of the Transmembrane Domain of SARS-CoV-2 Spike Protein in Detergent Micelles

**DOI:** 10.3390/ijms23031040

**Published:** 2022-01-18

**Authors:** Qingxin Li, Qiwei Huang, Congbao Kang

**Affiliations:** 1 Guangdong Provincial Engineering Laboratory of Biomass High Value Utilization, Institute of Biological and Medical Engineering, Guangdong Academy of Sciences, Guangzhou 510316, China; qingxin_li@outlook.com; 2 Experimental Drug Development Centre (EDDC), Agency for Science, Technology and Research (A*STAR), Singapore 138670, Singapore; huang_qiwei@eddc.a-star.edu.sg

**Keywords:** COVID-19, SARS-CoV-2, S-protein, transmembrane domain, NMR, detergent micelles

## Abstract

Spike protein of SARS-CoV-2 contains a single-span transmembrane (TM) domain and plays roles in receptor binding, viral attachment and viral entry to the host cells. The TM domain of spike protein is critical for viral infectivity. Herein, the TM domain of spike protein of SARS-CoV-2 was reconstituted in detergent micelles and subjected to structural analysis using solution NMR spectroscopy. The results demonstrate that the TM domain of the protein forms a helical structure in detergent micelles. An unstructured linker is identified between the TM helix and heptapeptide repeat 2 region. The linker is due to the proline residue at position 1213. Side chains of the three tryptophan residues preceding to and within the TM helix important for the function of S-protein might adopt multiple conformations which may be critical for their function. The side chain of W1212 was shown to be exposed to solvent and the side chains of residues W1214 and W1217 are buried in micelles. Relaxation study shows that the TM helix is rigid in solution while several residues have exchanges. The secondary structure and dynamics of the TM domain in this study provide insights into the function of the TM domain of spike protein.

## 1. Introduction

The epidemic of novel coronavirus disease (COVID-19) started in late 2019. COVID-19 was declared as a pandemic in March 2020 by the World Health Organization (WHO) [1,2,3]. The lifestyles of people from many countries were affected as this disease can be spread through close contact. This disease is caused by a novel coronavirus which is named as severe acute respiratory syndrome coronavirus 2 (SAR-CoV-2). SARS-CoV-2 belongs to beta-coronavirus which also contains other important human pathogens such as SARS-CoV which caused a viral outbreak in 2003 [4] and Middle East respiratory syndrome (MERS) coronavirus which was the cause of MERS in 2012 [5]. Efforts have been made to develop vaccines and antivirals, which plays important roles in preventing viral spread. The genome of SARS-CoV-2 is very similar to that of SARS-CoV [6] and is a single-strand and positive-sense RNA. The genome of the virus encodes 16 non-structural proteins (nsp1-16), 4 structural and 9 accessory proteins that are indispensable for viral replication, invasion and particle formation [6]. 

The viral lipid envelop contains three transmembrane proteins including spike (S), membrane (M) and envelope (E) proteins. S protein is a type-I transmembrane (TM) glycoprotein and functions as homotrimers on the viral surface [7]. S protein is highly conserved among human coronaviruses and the molecular weight of the protein is at the range of 180–200 kDa [8]. S protein is an important target for antiviral development as it plays important roles in binding to angiotensin-converting enzyme 2 (ACE2) receptors, viral attachment to and entry into host cells [7,9,10]. S protein of SARS-CoV-2 is composed of 1273 amino acids and contains a long external region, a TM domain and a short C-terminus [11]. S protein consists of several domains including an N-terminal signal peptide, an N-terminal domain, a receptor binding domain, a fusion peptide, heptapeptide repeats (HRs) 1 and 2, a TM domain and the cytoplasm domain [8]. The structures of the domains and the entire S protein have been studied using X-ray crystallography and Cryo-EM. These high-resolution structures provide clear information to understand its interaction with ACE2 and its conformational changes under different conditions [7,8,12]. The structural studies of S protein are critical for antiviral development and understanding its function. Strategies affecting S protein interactions with ACE2 such as developing antibodies have been explored in antiviral development [13,14].

The TM domain is close to heptad repeat 2 (HR2) and consists of a hydrophobic core region with a tryptophan-rich region and a cysteine-rich region at its N- and C-termini, respectively. The TM domain of S-protein is critical for viral infectivity and mutation of the tryptophan residues in the TM domain was found to decrease viral infectivity significantly [15]. A study shows that the TM domain of S protein is critical for the infectivity and membrane fusion activity of coronavirus [16]. Despite the progress made in structural studies of the N-terminal region of S-protein, only one structural study on the TM domain in bicelles was reported recently [17]. Here, we use solution nuclear magnetic resonance spectroscopy (NMR) to investigate the structure and dynamics of TM domain of S protein in detergent micelles. The current study uses a longer construct reconstituted in a different membrane system. The NMR studies will be helpful for choosing suitable membrane systems in structural studies of membrane proteins. Although detergent micelles may not be an ideal system for structural and functional studies of membrane proteins, they still serve as a useful membrane system to understand the secondary structure of a membrane protein. 

The TM domain of the S protein of SARS-CoV-2 was expressed and purified from *E. coli*. The purified protein was reconstituted into detergent micelles. Structural analysis using solution NMR spectroscopy shows that the TM contains an α-helix and an unstructured region is present between the helix from HR 2 and the TM domain. The indole amide proton of W1212 exhibited close contact with water molecules, suggesting that it is exposed to the solvent. Dynamics study shows that the TM helix is rigid in solution while Y1220 and L1224 might undergo exchanges. Our current study provides useful structural information to understand the function of the TM domain of S protein. 

## 2. Results

### 2.1. Solution NMR Spectrum of S-TM in Micelles

**Figure 1:**
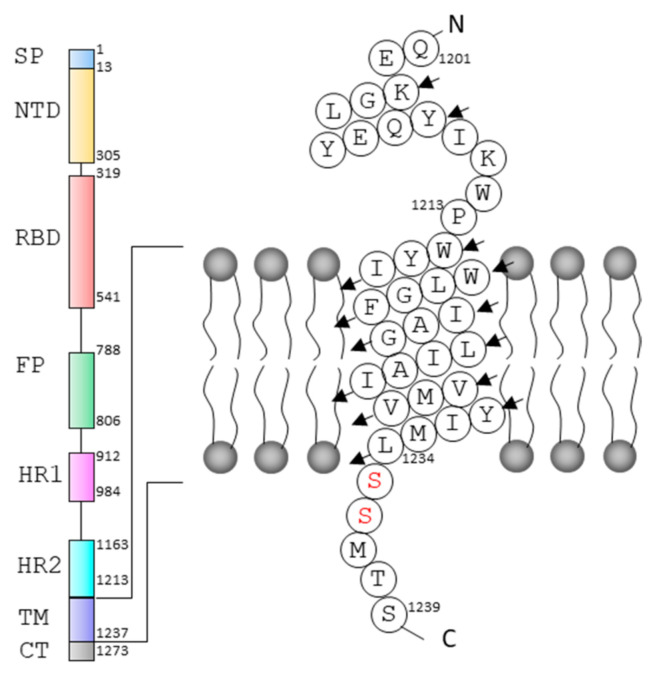
Sequence of S-TM used in this study. The domains of S protein of SARS-CoV-2. The model of S-TM in a membrane is shown. Two cysteine residues that are mutated into serine are highlighted in red. SP, signal peptide; NTD, N-terminal domain; RBD, receptor-binding domain; FP, fusion peptide; HR, heptapeptide repeat; TM, transmembrane domain; CT, C-terminal cytoplasmic domain. The N- and C-termini of the sequence are indicated as N and C, respectively.

**Figure 2:**
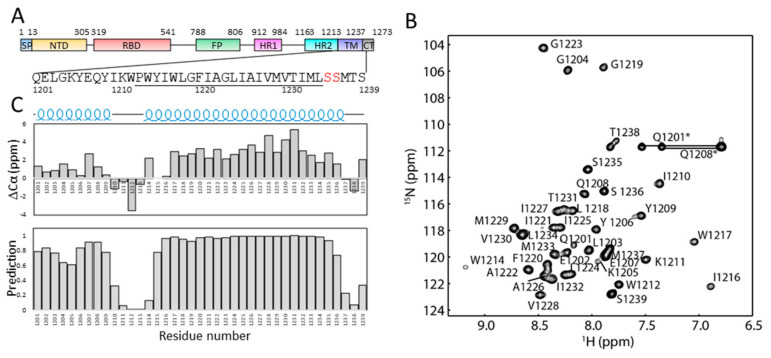
Secondary structure of S-TM in DPC micelles. (**A**). The sequence of S-TM used in this study. (**B**). Assignment of the ^1^H-^15^N-HSQC spectrum of S-TM in DPC micelles. The cross-peaks in the spectrum are labelled with a single letter and sequence number. (**C**). Secondary structure of S-TM in DPC micelles. The prediction of the secondary structures was obtained by analyzing Cα chemical shifts and chemical shifts using TALOS + Residues in the transmembrane domain are underlined. Cross-peaks corresponding to residues from the fusion tag and side chains of Q residues are labeled with “*”.

To understand the structure of the TM domain in solution, a construct containing residues 1201–1239 of SARS-CoV-2 S protein (S-TM) was obtained and expressed in *E. coli* (Figure S1). The recombinant protein contains an N-terminal fusion tag for aiding in affinity purification, a short stretch from HR2 (amino acids 1201–1213), the tryptophan-rich region, the hydrophobic region of the TM domain and several residues from the cysteine rich region (Figure 1). To prevent sample aggregation due to formation of disulfide bonds, the cysteine residues in the construct were mutated into serine residues (Figure 2A). The recombined protein was able to be purified from *E. coli* for structural studies. The S-TM reconstituted into dodecylphosphocholine (DPC) micelles was obtained and subjected to structural analysis using solution NMR spectroscopy. DPC is a widely used detergent in membrane protein structural studies and DPC micelles have been successful for structural studies of quite a few membrane proteins [18,19,20,21,22]. Dispersed cross-peaks were observed in the ^1^H-^15^N HSQC spectrum collected at 40 °C (Figure S2). Similar to other helical membrane proteins, S-TM exhibited narrow dispersion of the cross-peaks at the range of 6.8–9.2 ppm suggesting the presence of helical structures in this construct (Figure 2B).

### 2.2. Secondary Structures of S-TM

Backbone resonance assignment was obtained using conventional triple-resonance NMR experiments. Nearly complete assignments for the backbone atoms were obtained and the assignment has been deposited into BMRB under access number 51193. The secondary structures of residues from S-TM were then predicted based on the obtained chemical shifts of backbone resonances. Both chemical shift index analysis of Cα chemical shifts and TALOS+ analysis show that S-TM contains two helical segments (Figure 2C). Residues 1201–1209 from HR2 form a helix in solution. Residues from the hydrophobic core region of TM form a helix which contains residues 1215–1236. The three amino acids at the C-terminus of S-TM are not structured. Residues 1210–1215 between HR2 and the TM are unstructured serving as a linker region between HR2 and TM domain, which may be due to the presence of a proline amino acid at position 1213 (Figure 2A). The linker might provide freedom to the N-terminal region to alter its orientation under different conditions without affecting the structure of the TM domain significantly. 

### 2.3. Structural Model of S-TM

**Figure 3:**
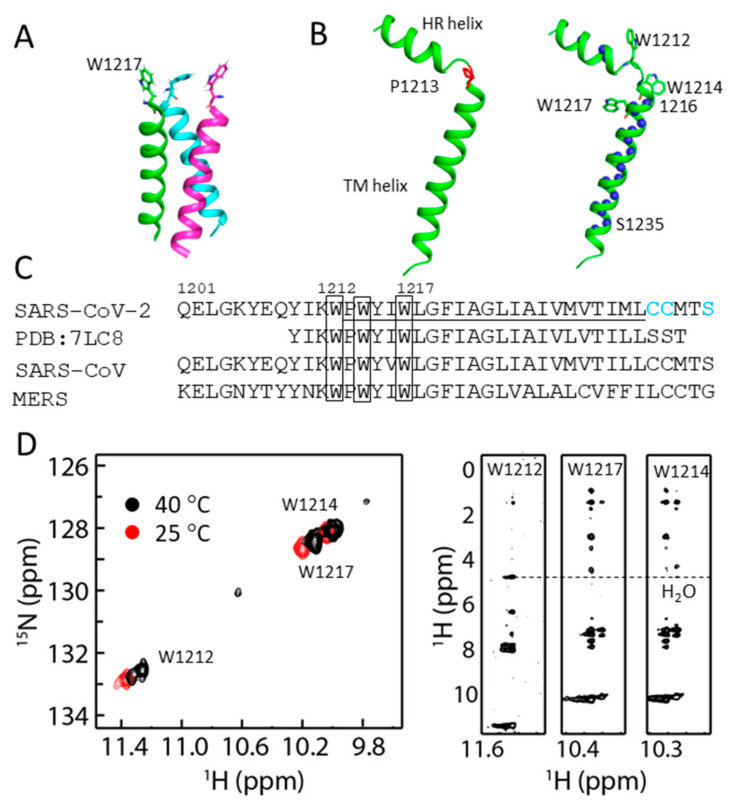
Structure of S-TM in DPC micelles. (**A**). Trimer structure of the TM domain of S protein determined in bicelles. The structure of TM in bicelles (PDB ID 7LC8) is shown [17]. W1217 in the structure is shown as sticks. (**B**). A structural model of S-TM in DPC micelles. The model was generated based on the backbone resonance assignment, H-D exchange experiment and a few NOE restraints. The orientations of the helices are not defined as no restraints are applied. Residues with cross-peaks in the HSQC spectrum of S-TM in D_2_O are shown as spheres. (**C**). Sequence alignment of current construct (QHD43416.1) with the available NMR structure, SARS-CoV (AAS75868.1), and MERS (QDI73610.1). Three serine residues in S-TM construct are highlighted in blue. (**D**). Analysis of the side chains of Trp residues in S-TM. Left panel, the ^1^H-^15^N-HSQC spectra of S-TM collected at different temperatures. Right panel, strip-plots of a ^15^N-edited NOESY spectrum for the side chains of Trp residues are shown. NOE between water molecules and indole amide proton of W1212 is indicated with a dashed line, suggesting that it is exposed to the solvent.

A ^1^H-^15^N HSQC spectrum of S-TM in D_2_O was collected to understand secondary structure and dynamics of S-TM in DPC micelles. Three residues from the HR helix including Y1206, Y1209 and I1210 exhibited cross-peaks in the spectrum. These residues should be involved in hydrogen bond formation confirming their helical structure in solution. Residues W1217 to S1235 from the TM helix exhibited cross-peaks in the spectrum (Figure S3). These residues having cross-peaks in the HSQC spectrum suggests their participation in hydrogen bond formation or that they are buried in micelles. The structure of the TM domain of S protein in bicelles was determined recently [17] (Figure 3A). Our study also shows that residues 1214 to 1216 form helical structure in solution. A structural model was built based on the secondary structure analysis and the hydrogen-deuterium exchange experiment (Figure 3B). The chemical shift analysis (Figure 3C), H/D exchange experiment, and lack of long-range distance restraints strongly suggest that P1213 is a helix breaker in S-TM.

### 2.4. Conformational Analysis of Residues in S-TM

There are three tryptophan residues in S-TM and these residues are conserved in SARS-CoV and MERS S proteins (Figure 3C). We explored the conformational status of these residues by overserving the signals from the indole ring as they appear in a distinct region of the ^1^H-^15^N-HSQC spectrum (Figure 3C). Three peaks corresponding to the side chains of tryptophan residues were observed at 25 °C while more peaks were observed at 40 °C for W1212. The appearance of multiple peaks of W1212 in the spectrum suggests that its side chains might adopt multiple conformations in DPC micelles or are located in different environments [23]. It is obvious that the side chain of W1212 is exposed to the solvent as it exhibited close contact with water molecules, evidenced by NOE with water molecules identified in the NOESY spectrum (Figure 3D). The side chains of W1214 and W1217 are buried in micelles as no NOEs were identified in the spectrum (Figure 3D). Interestingly, the cross-peak of amide and amide proton for W1214 in the HSQC spectrum is broadened compared with that of W1217 (Figure 2B). Such a difference suggests that there might be exchanges for residue W1214. Taking together, W1212 localizes in the linker between these two helices, giving rise to multiple conformations. W1214 localizes at the interface of cell membrane with its side chain buried in the membrane, and conformational exchanges may exist to result in line broadening in the spectrum (Figure 3D). W1217 is buried in the membrane and may play important roles in stabilizing the structure of the transmembrane region. Further mutation studies on these residues will be helpful for understanding their roles. 

### 2.5. Dynamics of S-TM

**Figure 4:**
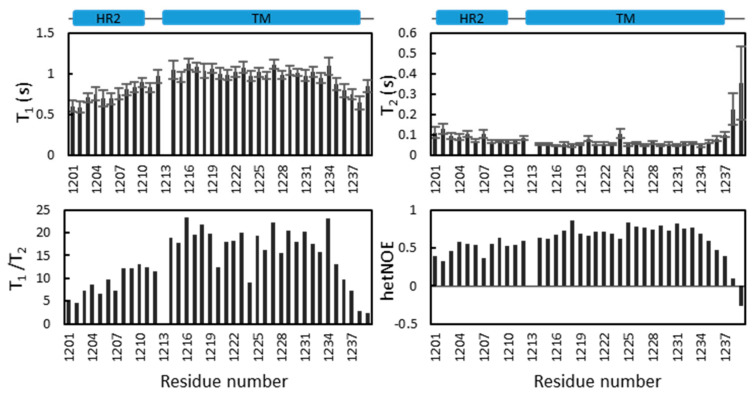
^15^N-T_1_, T_2_ and hetNOE analysis of S-TM in DPC micelles. The data were collected on a Bruker Avance 600 MHz spectrometer equipped with a cryo-probe. ^15^N-T_1_, T_2_ and hetNOE values of S-TM are plotted against sequence number. The secondary structures of HR2 and TM domain are indicated as blue boxes.

The dynamics of S-TM in DPC micelles were investigated by measuring ^15^N-T_1_ (spin–lattice relaxation), T_2_ (spin–spin relaxation) and steady-state ^1^H-^15^N NOE values (Figure 4). The data offer additional information relevant to understanding the structure of S-TM in DPC micelles. The two helices are rigid while the linker between these two helices is flexible. Such flexibility might be important for the function of S-protein. The T_1_ values of residues from the helix in HR2 are lower than those of residues in the TM region, which is not surprising as residues in the TM region are buried in DPC micelles. A correlation time of approximately 13 ns was estimated based on the average T_1_/T_2_ value for residues in the TM region [20], which suggests that S-TM under current conditions is monomeric. Further dynamics study of S-TM in different membrane systems and data acquisition under different magnetic fields will be helpful for understanding its dynamics. In addition, analyzing the simulated relaxation rates based on the structures will be very helpful for determining the oligomeric states of the sample and identifying changes in different time scales [24].

To detect whether S-TM in DPC can form oligomers we performed a cross-linking study using glutaraldehyde [25]. In the absence of the cross linker, S-TM exhibited a band at the molecular weight of 10 kDa which is above its molecular weight (~7 kDa). Such difference may be due to the presence of DPC micelles in the sample. A band corresponding to a higher molecular weight (~14 kDa) was observed in SDS-PAGE (Figure S4), demonstrating that the construct in DPC micelles can form dimers under such conditions. Further optimization of the experimental conditions is needed to obtain trimeric S-TM. The T_1_/T_2_ values of residues in the HR2 helix are lower than those of residues in the TM domain, suggesting that the HR2 helix does not have interactions with DPC micelles. This is consistent with the fact that folding of HR2 does not require the presence of membrane systems. Residues including Y1220 and L1224 exhibited lower T_1_/T_2_ values than those in the transmembrane helix. These residues may undergo exchanges under current conditions.

## 3. Discussion

SARS-CoV-2 spike protein is an important target for antiviral development [26]. The structures of spike protein have been investigated by different methods, providing critical information for developing antivirals. Only one study was carried out to explore the structure of the TM domain of spike protein in bicelles using NMR spectroscopy to provide the structural basis for trimer formation [17]. In the current study, we obtained S-TM in detergent micelles. The secondary structure of S-TM in micelles was obtained based on the chemical shifts and an H/D exchange experiment (Figure 2 and Figure 3). The TM domain exists as a helical structure in detergent micelles, which is same as in bicelles. The TM forms a rigid structure in both bicelles and DPC micelles, while exchanges were observed for some residues in micelles. This may be due to the monomeric structure in the current study. In addition, we have identified a linker region formed by residues 1211 to 1213 between TM and HR2. Proline 1213 is critical for formation of the linker between the HR2 and TM helices (Figure 3). The presence of the linker might be critical for the function of S protein under different conditions.

The tryptophan residues are critical for the function of spike protein and mutations of the residues in the TM domain were found to have an impact on viral infectivity [15]. We demonstrate that the side chain of W1212 is exposed to the solvent and exhibits conformational changes under different conditions. W1212 localizes at the linker between HR2 and TM domain. W1214 is close to the water and membrane interface and W1217 is within the transmembrane region. No NOEs with water molecules were observed, suggesting that the side chains of W1214 and W1217 are buried in the micelles. Relaxation analysis also support this conclusion (Figure 4). W1212 exhibited similar dynamics parameters to those in the HR2 helix while W1214 and W1217 behave similarly to those residues in the transmembrane helix (Figure 4). It has been noted that the side chain of a tryptophan is critical for the stability and orientation of a transmembrane protein [27]. Our study provides insights into the location of these residues, which will be useful for interpreting their functional roles in viral infectivity. Further mutations can be made to elucidate the effect of these residues on protein structure and dynamics. 

The S-TM in the current study exists as monomers in DPC micelles as evidenced by the relaxation analysis (Figure 4), and the cross-linking experiment shows it can form dimers in the presence of the cross linker (Figure S4). The TM domain of S protein is functional as trimers [28] and a recent study by Chou’s team showed that the TM domain of S protein exists as a strong trimer in bicelles [17]. The current S-TM construct in DPC micelles did not form functional trimers, which may be due to the following aspects. First, the fusion tag was not cleaved after protein purification. The presence of fusion tag may affect the formation of oligomers. Second, DPC was used in the current study. It is known that detergent micelles might not be the ideal system for exploring the structures of membrane proteins [29,30,31] although several structures of membrane proteins have been determined in DPC micelles [18,32,33]. Optimization of the experimental conditions such as the ratio of DPC to S-TM needs will be helpful to obtain functional trimers in solution. Finally, the M1229 in the current construct might affect trimer formation. A previous study showed that L1229Y mutation disrupted trimer formation as it is part of the hydrophobic core [17]. Further optimization of the conditions will be helpful for obtaining S-TM trimers. Although detergent micelles might not be an ideal system for structural study of a membrane protein, this study provides secondary structural information to understand the roles of this domain.

## 4. Materials and Methods

### 4.1. Protein Expression and Purification

The cDNA encoding residues 1201–1239 of SARS-CoV-2 S protein (S-TM) was synthesized and cloned into pET15b. The resulting plasmid encodes a recombinant protein containing a fusion tag and thrombin cleavage site to remove the fusion tag containing the following amino acids MGSSHHHHHHSSGLVPRGS. The plasmid was transformed into *Escherichia coli* (*E. coli*) BL21 (DE3) competent cells from Stratagene (La Jolla, CA, USA), which were grown in M9 medium supplied with 100 µg/mL ampicillin. When OD_600_ reached 0.6–0.8, protein induction was initiated by adding β-D-1-thiogalactopyranoside (IPTG) to 1 mM and the cells were further cultured at 37 °C and 200 rpm overnight. The recombinant protein was purified into detergent micelles as described previously [20,34].

The *E. coli* cells with recombinant S-TM were harvested by centrifugation at 9000× *g* for 10 min. The cell pellet was suspended into a lysis buffer (20 mM Tris-HCl, 300 mM NaCl, pH 7.8, and 2 mM β-mercaptoethanol) and cells were lysed by sonication. Inclusion bodies were obtained by centrifugation at 18,000× *g* for 20 min. The inclusion bodies were washed with the lysis buffer and suspended in a urea buffer (8 M urea, 300 mM NaCl, 10 mM SDS, 20 mM Tris-HCl, pH 7.8). The solution was cleared by centrifugation at 48,000× *g* for 20 min. The supernatant was mixed with nitrilotriacetic acid saturated with nickel (Ni^2+^-NTA) resin from Qiagen (Gmbh, Germany) which was loaded in a gravity column. The resin was washed with a washing buffer (8 M urea, 300 mM NaCl, 10 mM SDS, 20 mM Tris-HCl, pH 7.8 and 20 mM imidazole). The resin was then washed with washing buffer 2 (20 mM Tris-HCl, pH 7.8, 300 mM NaCl and 10 mM SDS) to remove urea. To reconstitute the protein in DPC micelles, the resin was washed with washing buffer 3 (20 mM Tris-HCl, pH 7.8, 300 mM NaCl and 15 mM DPC). Recombinant protein was eluted using an elution buffer (300 mM imidazole, pH 6.5 and 15 mM DPC). Purified protein was then further purified through gel filtration using a gel filtration buffer (20 mM sodium phosphate, pH 6.5 and 15 mM DPC) on a superdex^TM^ 200 10/300 GL column. The sample was then combined and concentrated to 1 mM and the concentration of DPC was estimated to 150 mM. IPTG, DTT and detergents used in the study were purchased from Anatrace (Maumee, OH, USA) or Avanti Polar Lipids (Birmingham, AL, USA). The ^15^NH_4_Cl, ^13^C-glucose and D2O were obtained from Cambridge Isotope Laboratories (Andover, MA, USA). All other chemicals used in this study were purchased from Sigma–Aldrich. 

### 4.2. Resonance Assignment

All the NMR spectra were collected at 40 °C to gain signals in the spectra for resonance assignment. The experiments were carried out on a Bruker Avance spectrometer (Bruker, Germany) with a proton frequency of 600 MHz and equipped with a cryogenic triple-resonance probe. Data were acquired using Topspin 2.1 and were processed with NMRPipe [35] and analyzed using NMRView [36]. Sequence-specific assignments of backbone resonances were obtained based on triple-resonance experiments using a ^15^N/^13^C-labeled S-TM in DPC micelles. These experiments include HNCACB, HN(CA)CO, HNCA, HN(CO)CACB, HN(CO)CA, and HNCO. The chemical shifts of Hα and Hβ were assigned using a HBHA(CO)NH experiment. Secondary structure of S-TM in DPC micelles was identified by analysis of ^13^Cα chemical shifts [37] and TALOS+ [38]. A 3D ^15^N-edited NOESY (mixing time = 100 ms) was collected and some peaks were manually assigned and the peak intensities were converted into distance restraints using CYANA [39]. The restraints include dihedral angles, hydrogen bonds derived from a H/D exchange experiment and NOE distance restraints [40,41,42]. In total, one hundred structural models were built, and one structure was selected as the model to understand the structure of S-TM in DPC micelles. More restraints will be required for determining the orientation of the two helices in S-TM.

### 4.3. Collection of the ^1^H-^15^N HSQC Spectrum in D_2_O

The hydrogen-deuterium exchange experiment was performed to identify residues that form hydrogen bonds. Recombinant S-TM was first purified into DPC micelles as previously described. The sample was then frozen in liquid nitrogen. After removing water from the sample through lyophilization, 99.9% D_2_O was added into the sample. The sample was then subject to data acquisition. The acquisition time of the ^1^H-^15^N HSQC spectrum took approximately 10 min. The data was then processed and visualized. Residues that exhibited cross peaks in the ^1^H-^15^N HSQC spectrum are involved in hydrogen bond formation or buried deeply in the micelles. 

### 4.4. Relaxation Analysis

T_1_, T_2_ and ^1^H-^15^N steady-state NOE values [43] were obtained by collecting the data acquired at 313 K on a Bruker Avance 600 MHz spectrometer. For T_1_ and T_2_ measurements, pseudo-3D experiments with different delays were collected and processed as described previously [44,45,46]. Steady-state ^1^H-^15^N NOEs (hetNOE) were calculated by analyzing two datasets that were collected with and without initial proton saturation for a period of 3 s [47].

### 4.5. Cross-Linking Experiment

A cross-linking experiment using glutaraldehyde (GA) was performed as previously described [20,21,34,46]. Briefly, the mixture contained 25 µM S-TM in a buffer containing 20 mM sodium phosphate, pH 6.5 and 15 mM DPC. GA was added to 16 mM concentration. The mixture was kept and the samples at different time points were collected and mixed with SDS loading dye. The samples were then subjected to analysis by SDS-PAGE.

## 5. Conclusions

The structure of S-TM in detergent micelles was explored. A flexible linker was identified between HR2 and the TM helix. The structure and dynamics of S-TM in DPC micelles show that the TM domain adopts a helix, and some residues might undergo exchanges. The current study provides structural information to assist in understanding the function of S-protein.

## Data Availability

The resonance assignment has been deposited into BMRB under access number 51193.

## Supplementary Materials

The following supporting information can be downloaded at: https://www.mdpi.com/article/10.3390/ijms23031040/s1.

## References

[1] Hui D.S., Azhar E.I., Madani T.A., Ntoumi F., Kock R., Dar O., Ippolito G., McHugh T.D., Memish Z.A., Drosten C. (2020). The continuing 2019-nCoV epidemic threat of novel coronaviruses to global health—The latest 2019 novel coronavirus outbreak in Wuhan, China. Int. J. Infect. Dis. IJID Off. Publ. Int. Soc. Infect. Dis..

[2] Kaul D. (2020). An overview of coronaviruses including the SARS-2 coronavirus—Molecular biology, epidemiology and clinical implications. Curr. Med. Res. Pract..

[3] Zhu N., Zhang D., Wang W., Li X., Yang B., Song J., Zhao X., Huang B., Shi W., Lu R. (2020). A novel coronavirus from patients with pneumonia in China, 2019. N. Engl. J. Med..

[4] Lipsitch M., Cohen T., Cooper B., Robins J.M., Ma S., James L., Gopalakrishna G., Chew S.K., Tan C.C., Samore M.H. (2003). Transmission dynamics and control of severe acute respiratory syndrome. Science.

[5] Sharif-Yakan A., Kanj S.S. (2014). Emergence of MERS-CoV in the Middle East: Origins, transmission, treatment, and perspectives. PLoS Pathog..

[6] Zhou P., Yang X.L., Wang X.G., Hu B., Zhang L., Zhang W., Si H.R., Zhu Y., Li B., Huang C.L. (2020). A pneumonia outbreak associated with a new coronavirus of probable bat origin. Nature.

[7] Walls A.C., Park Y.J., Tortorici M.A., Wall A., McGuire A.T., Veesler D. (2020). Structure, function, and antigenicity of the SARS-CoV-2 spike glycoprotein. Cell.

[8] Huang Y., Yang C., Xu X.-F., Xu W., Liu S.-W. (2020). Structural and functional properties of SARS-CoV-2 spike protein: Potential antivirus drug development for COVID-19. Acta Pharmacol. Sin..

[9] Yan R., Zhang Y., Li Y., Xia L., Guo Y., Zhou Q. (2020). Structural basis for the recognition of SARS-CoV-2 by full-length human ACE2. Science.

[10] Tai W., He L., Zhang X., Pu J., Voronin D., Jiang S., Zhou Y., Du L. (2020). Characterization of the receptor-binding domain (RBD) of 2019 novel coronavirus: Implication for development of RBD protein as a viral attachment inhibitor and vaccine. Cell. Mol. Immunol..

[11] Yang T.-J., Yu P.-Y., Chang Y.-C., Hsu S.-T.D. (2021). D614G mutation in the SARS-CoV-2 spike protein enhances viral fitness by desensitizing it to temperature-dependent denaturation. J. Biol. Chem..

[12] Tian X., Li C., Huang A., Xia S., Lu S., Shi Z., Lu L., Jiang S., Yang Z., Wu Y. (2020). Potent binding of 2019 novel coronavirus spike protein by a SARS coronavirus-specific human monoclonal antibody. Emerg. Microbes Infect..

[13] Cao Z., Liu L., Du L., Zhang C., Jiang S., Li T., He Y. (2010). Potent and persistent antibody responses against the receptor-binding domain of SARS-CoV spike protein in recovered patients. Virol. J..

[14] Hussain A., Hasan A., Nejadi Babadaei M.M., Bloukh S.H., Chowdhury M.E.H., Sharifi M., Haghighat S., Falahati M. (2020). Targeting SARS-CoV2 spike protein receptor binding domain by therapeutic antibodies. Biomed. Pharmacother..

[15] Lu Y., Neo T.L., Liu D.X., Tam J.P. (2008). Importance of SARS-CoV spike protein Trp-rich region in viral infectivity. Biochem. Biophys. Res. Commun..

[16] Broer R., Boson B., Spaan W., Cosset F.-L., Corver J. (2006). Important role for the transmembrane domain of severe acute respiratory syndrome Coronavirus spike protein during entry. J. Virol..

[17] Fu Q., Chou J.J. (2021). A Trimeric hydrophobic zipper mediates the intramembrane assembly of SARS-CoV-2 spike. J. Am. Chem. Soc..

[18] Van Horn W.D., Kim H.-J., Ellis C.D., Hadziselimovic A., Sulistijo E.S., Karra M.D., Tian C., Sönnichsen F.D., Sanders C.R. (2009). Solution nuclear magnetic resonance structure of membrane-integral diacylglycerol kinase. Science.

[19] Yang Q., Brüschweiler S., Zhao L., Chou J.J. (2018). Reply to ‘Concerns with yeast mitochondrial ADP/ATP carrier’s integrity in DPC’ and ‘Dynamics and interactions of AAC3 in DPC are not functionally relevant’. Nat. Struct. Mol. Biol..

[20] Li Q., Ng H.Q., Kang C. (2019). Secondary structure and topology of the transmembrane domain of Syndecan-2 in detergent micelles. FEBS Lett..

[21] Li Y., Lee M.Y., Loh Y.R., Kang C. (2018). Secondary structure and membrane topology of dengue virus NS4A protein in micelles. Biochim. Biophys. Acta (BBA)-Biomembr..

[22] Li Q., Wong Y.L., Huang Q., Kang C. (2014). Structural insight into the transmembrane domain and the juxtamembrane region of the erythropoietin receptor in micelles. Biophys. J..

[23] Tifrea D.F., Pal S., Popot J.-L., Cocco M.J., de la Maza L.M. (2014). Increased immunoaccessibility of MOMP epitopes in a vaccine formulated with amphipols may account for the very robust protection elicited against a vaginal challenge with *Chlamydia muridarum*. J. Immunol..

[24] de la Torre J.G., Huertas M., Carrasco B. (2000). HYDRONMR: Prediction of NMR relaxation of globular proteins from atomic-level structures and hydrodynamic calculations. J. Magn. Reson..

[25] Vinogradova O., Badola P., Czerski L., Sonnichsen F., Sanders C. (1997). Escherichia coli diacylglycerol kinase: A case study in the application of solution NMR methods to an integral membrane protein. Biophys. J..

[26] Wolfe M., Webb S., Chushak Y., Krabacher R., Liu Y., Swami N., Harbaugh S., Chávez J. (2021). A high-throughput pipeline for design and selection of peptides targeting the SARS-Cov-2 Spike protein. Sci. Rep..

[27] de Jesus A.J., Allen T.W. (2013). The role of tryptophan side chains in membrane protein anchoring and hydrophobic mismatch. Biochim. Biophys. Acta (BBA)-Biomembr..

[28] Azad T., Singaravelu R., Crupi M.J.F., Jamieson T., Dave J., Brown E.E.F., Rezaei R., Taha Z., Boulton S., Martin N.T. (2020). Implications for SARS-CoV-2 vaccine design: Fusion of Spike Glycoprotein Transmembrane Domain To Receptor-Binding Domain Induces Trimerization. Membranes.

[29] Chipot C., Dehez F., Schnell J.R., Zitzmann N., Pebay-Peyroula E., Catoire L.J., Miroux B., Kunji E., Veglia G., Cross T.A. (2018). Perturbations of native membrane protein structure in Alkyl Phosphocholine detergents: A critical assessment of NMR and biophysical studies. Chem. Rev..

[30] Kang C., Vanoye C.G., Welch R.C., Van Horn W.D., Sanders C.R. (2010). Functional delivery of a membrane protein into oocyte membranes using Bicelles. Biochemistry.

[31] Coey A.T., Sahu I.D., Gunasekera T.S., Troxel K.R., Hawn J.M., Swartz M.S., Wickenheiser M.R., Reid R.-J., Welch R.C., Vanoye C.G. (2011). Reconstitution of KCNE1 into lipid bilayers: Comparing the structural, dynamic, and activity differences in micelle and vesicle environments. Biochemistry.

[32] Yeo K.J., Kim H.-Y., Kim Y.P., Hwang E., Kim M.H., Cheong C., Choe S., Jeon Y.H. (2010). Rapid exploration of the folding topology of helical membrane proteins using paramagnetic perturbation. Protein Sci..

[33] Lakomek N.-A., Kaufman J.D., Stahl S.J., Wingfield P.T. (2014). HIV-1 Envelope protein gp41: An NMR study of dodecyl phosphocholine embedded gp41 reveals a dynamic prefusion intermediate conformation. Structure.

[34] Li Q., Wong Y.L., Kang C. (2014). Solution structure of the transmembrane domain of the insulin receptor in detergent micelles. Biochim. Biophys. Acta (BBA)-Biomembr..

[35] Delaglio F., Grzesiek S., Vuister G.W., Zhu G., Pfeifer J., Bax A. (1995). NMRPipe: A multidimensional spectral processing system based on UNIX pipes. J. Biomol. NMR.

[36] Johnson B.A. (2004). Using NMRView to visualize and analyze the NMR spectra of macromolecules. Protein NMR Techniques.

[37] Wishart D.S., Sykes B.D., Richards F.M. (1992). The chemical shift index: A fast and simple method for the assignment of protein secondary structure through NMR spectroscopy. Biochemistry.

[38] Shen Y., Delaglio F., Cornilescu G., Bax A. (2009). TALOS+: A hybrid method for predicting protein backbone torsion angles from NMR chemical shifts. J. Biomol. NMR.

[39] Güntert P. (2004). Automated NMR Structure Calculation With CYANA. Methods Mol. Biol..

[40] Li Y., Ng H.Q., Li Q., Kang C. (2016). Structure of the Cyclic Nucleotide-Binding Homology Domain of the hERG Channel and Its Insight into Type 2 Long QT Syndrome. Sci. Rep..

[41] Li Q., Wong Y.L., Lee M.Y., Li Y., Kang C. (2015). Solution structure of the transmembrane domain of the mouse erythropoietin receptor in detergent micelles. Sci. Rep..

[42] Li Q., Ng H.Q., Yoon H.S., Kang C. (2014). Solution structure of the cyclic-nucleotide binding homology domain of a KCNH channel. J. Struct. Biol..

[43] Kay L.E., Torchia D.A., Bax A. (1989). Backbone dynamics of proteins as studied by 15N inverse detected heteronuclear NMR spectroscopy: Application to staphylococcal nuclease. Biochemistry.

[44] Li Y., Wong Y.L., Lee M.Y., Li Q., Wang Q.Y., Lescar J., Shi P.Y., Kang C. (2016). Secondary Structure and membrane topology of the full-length dengue virus NS4B in micelles. Angew. Chem. Int. Ed. Engl..

[45] Li Y., Li Q., Wong Y.L., Liew L.S.Y., Kang C. (2015). Membrane topology of NS2B of dengue virus revealed by NMR spectroscopy. Biochim. Biophys. Acta (BBA)-Biomembr..

[46] Li Y., Kim Y.M., Zou J., Wang Q.Y., Gayen S., Wong Y.L., Lee le T., Xie X., Huang Q., Lescar J. (2015). Secondary structure and membrane topology of dengue virus NS4B N-terminal 125 amino acids. Biochim. Biophys Acta.

[47] Gayen S., Li Q., Chen A.S., Nguyen T.H.T., Huang Q., Hill J., Kang C. (2011). An NMR study of the N-terminal domain of wild-type hERG and a T65P trafficking deficient hERG mutant. Proteins Struct. Funct. Bioinform..

