# Supplementary material for: Secondary Structures of the Transmembrane Domain of SARS-CoV-2 Spike Protein in Detergent Micelles
Source: Int J Mol Sci. 2022 Jan 18;23(3):1040. doi: 10.3390/ijms23031040 (PMC8834715; doi:10.3390/ijms23031040)
Supplement: Supplementary file 1 [file ijms-23-01040-s001.zip › ijms-1543806-supplementary.pdf]

Supplementary information

Secondary structures of the transmembrane domain of SARS-CoV-2 spike protein in detergent micelles

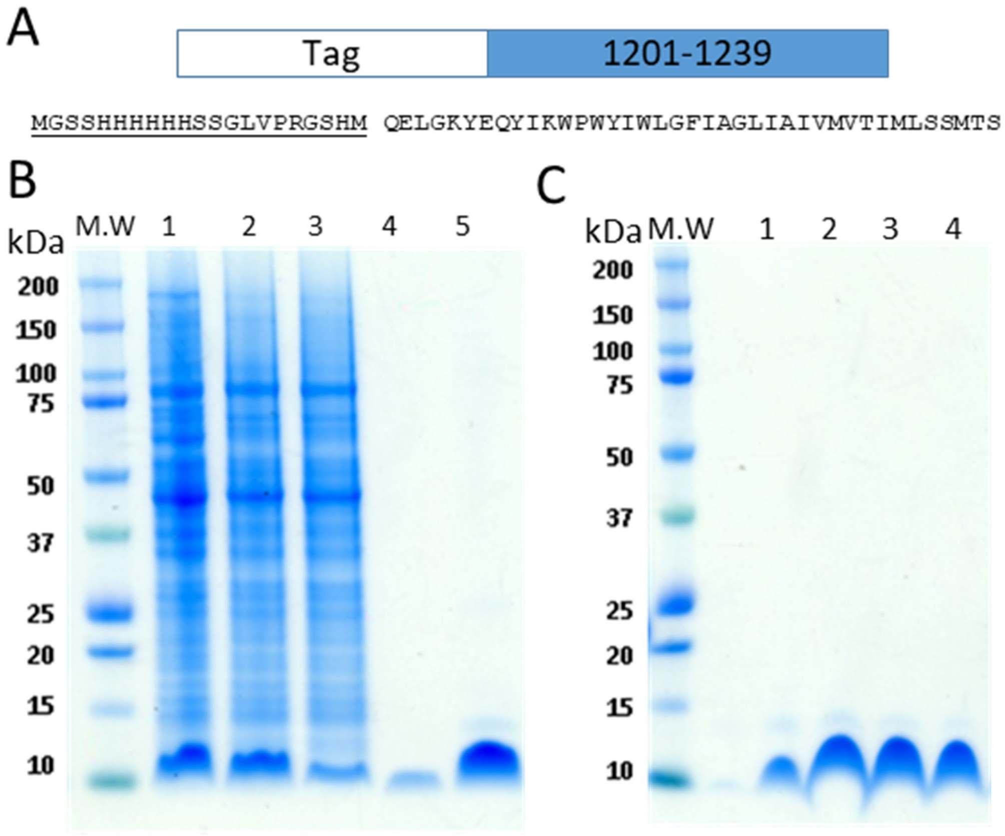

Figure S1 Purification of S-TM for structural studies. A. The constructs used in the study. A construct containing a tag at the N-terminus was used in the study. B. Purification of S-TM using Ni^{2+}-NTA resin. Lane 1-3 are total cell lysate, cell pellet after adding a urea buffer and flow through fraction from the resin, respectively. Lanes 4 and 5 are the eluted fraction from the resin. C. Purification of S-TM using gel filtration chromatography. Lanes 1 and 4 are the fractions containing S-TM protein.

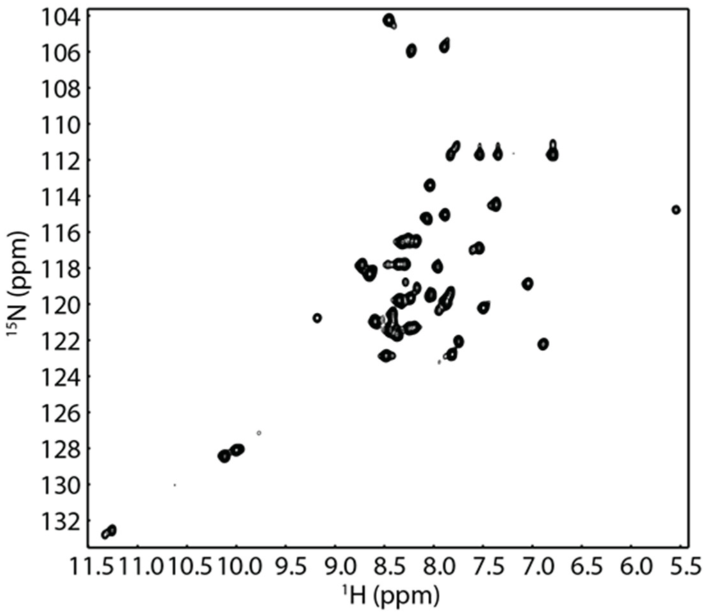

Figure S2 ^{1}H-^{15}N-HSQC spectrum of S-TM in DPC micelles. The data was collected 313K as described in the Materials and Methods.

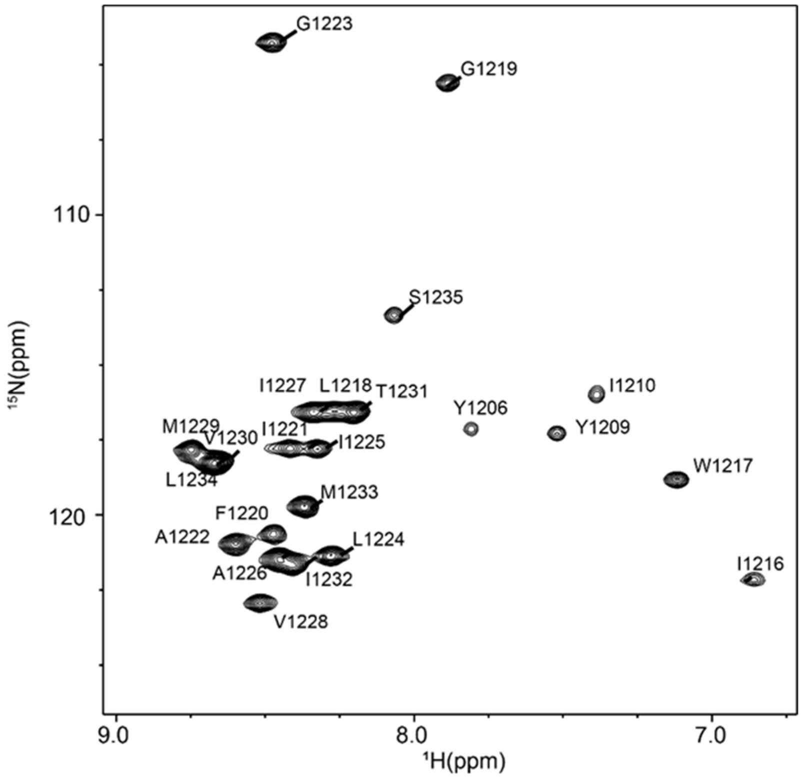

Figure S3 ^1H-^{15}N-HSQC spectrum of S-TM reconstituted in DPC micelles and in D_2O. Purified S-TM was lyophilized and 99% D2O was added into the sample. The ^1H-^{15}N-HSQC was collected after 10 min.

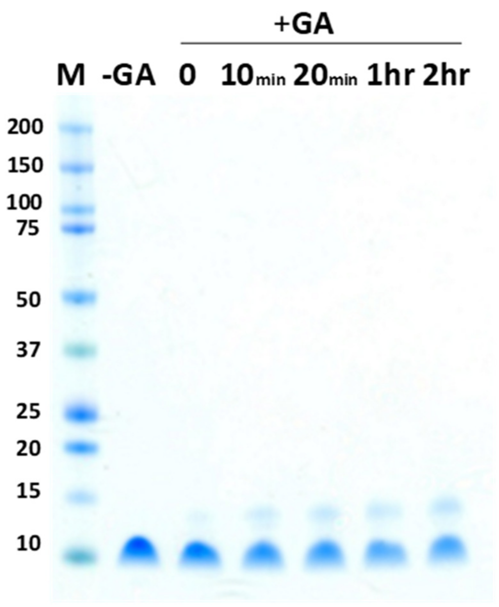

Figure S4 Cross-linking of S-TM in DPC micelles using glutaraldehyde (GA). The protein sample was mixed with GA as described in Materials and Methods and then subjected to analysis by SDS-PAGE.
